# The Role of Serotonin in Sleep Disordered Breathing Associated with Parkinson Disease: A Correlative [^11^C]DASB PET Imaging Study

**DOI:** 10.1371/journal.pone.0040166

**Published:** 2012-07-05

**Authors:** Irene M. Lelieveld, Martijn L. T. M. Müller, Nicolaas I. Bohnen, Robert A. Koeppe, Ronald D. Chervin, Kirk A. Frey, Roger L. Albin

**Affiliations:** 1 Faculty of Psychology and Neuroscience, Maastricht University, Maastricht, The Netherlands; 2 Department of Radiology, University of Michigan, Ann Arbor, Michigan, United States of America; 3 Department of Neurology, University of Michigan, Ann Arbor, Michigan, United States of America; 4 Geriatrics Research, Education, and Clinical Center, VAAAHS, Ann Arbor, Michigan, United States of America; 5 Sleep Disorders Center, University of Michigan, Ann Arbor, Michigan, United States of America; The Chinese University of Hong Kong, Hong Kong

## Abstract

Sleep dysfunction and excessive daytime sleepiness are common in Parkinson disease (PD). Several studies suggest that PD patients exhibit high prevalence of sleep-disordered breathing (SDB). PD has a complex profile of neurochemical deficits in which abnormalities of different neurotransmitter systems may play significant and differing roles in the development of non-motor features. In the present study, we investigated whether SDB in PD is related to serotoninergic neuron degeneration. We used a cross-sectional design to assess the correlation between SDB and measures of caudal brainstem serotonin neuron integrity. Fifty one PD participants with mean disease duration of 6.0 (SD 3.7) years and mean age of 63.9 (SD 6.2) years were studied. We measured caudal brainstem serotoninergic innervation with [^11^C]DASB positron emission tomography (PET) imaging and striatal dopaminergic innervation with [^11^C]DTBZ PET imaging. SDB was assessed with polysomnography (PSG) and sleepiness with multiple sleep latency tests. Greater than half of participants exhibited PSG evidence of significant SDB; 12 participants had normal PSGs, 6 had mild SDB, 20 had moderate SDB, and 13 had severe SDB. We found no association between severity of SDB and caudal brainstem serotoninergic innervation in PD participants. Striatal dopaminergic denervation did not correlate with severity of SDB. We did find significant correlations between measures of motor function impairment and sleep quantity and quality in PD. Neither serotoninergic nor dopaminergic neuron degeneration is likely to play a major role in SDB observed in PD patients.

## Introduction

Parkinson disease (PD) is characterized by several different motor and non-motor features. The cardinal motor symptoms of PD include tremor, rigidity, bradykinesia/akinesia, and postural instability. Among the common non-motor features of PD are sleep disturbances, cognitive impairment, depression, anxiety, apathy, and hyposmia/anosmia [1]–[7]. Sleep disorders and excessive daytime sleepiness have a major effect on quality of life in PD [8]–[10]. An important sleep disorder described in several recent studies of PD is sleep-disordered breathing (SDB; obstructive sleep apnea). Several cross-sectional studies using polysomnographic (PSG) criteria for SDB describe a range of elevated SDB prevalence in PD with 20%–67% of PD participants described as exhibiting SDB [11]–[16].

In SDB, control of pharyngeal dilator muscles maintaining upper airway patency is disturbed, causing airway dysfunction during sleep. While asleep, there is diminished muscle tone of the tongue, resulting in airway obstruction in patients more prone to SDB, especially patients with an increased body mass index (BMI) [17]. Pharyngeal dilator muscles are innervated by brainstem motor neurons modulated by several neurotransmitter systems. One neurotransmitter involved in excitation of these motor neurons is serotonin [18]. The serotoninergic neurons of the caudal brainstem, those of the caudal pons and medulla, innervate the pharyngeal dilator motoneurons and their integrity and function may be important for control of upper airway patency during sleep [19]. Caudal brainstem serotoninergic neurons are important also for maintaining normal rhythmic respiration [20].

A recent study of individuals with a history of abuse of the selective serotoninergic neurotoxin MDMA (‘ecstacy’) supports a role for serotoninergic system abnormalities in SDB. MDMA abusers demonstrated increased risk for SDB with greater lifetime MDMA exposure correlating with increased rates of SDB [21]. Experimental animal and human genetic studies also suggest a role for altered serotoninergic neuron function in the pathogenesis of SDB [22], [23].

The neuropathological hallmark of PD is neurodegeneration of substantia nigra pars compacta dopaminergic neurons projecting to the striatum with accumulation of cytoplasmic Lewy bodies in surviving neurons [24], [25]. Degeneration of other neuromodulatory systems is documented as well, including serotoninergic systems [26]. Significant reductions of serotonin transporter (SERT) binding, a measure of serotoninergic terminals, are reported in several brain areas of PD participants with positron emission tomography (PET) using the selective SERT ligand [^11^C]-3-amino-4-(2-dimethylaminomethyl-phenylsulfanyl)-benzonitrile ([^11^C]DASB) [27], [28]. In a more recent *in vivo* DASB PET study, Albin *et al.* (2008) found diminished SERT expression (approximately 20%–35% of controls) in rostral and caudal brainstem structures of PD participants and similar changes are reported recently by Politis *et al.* [29], [30]. The clinical significance of degeneration of serotoninergic systems and their contribution to non-motor clinical features of PD are largely unexplored. The conjunction of serotoninergic degeneration in PD and prior work on serotoninergic regulation of upper airway function and respiration suggests that abnormalities of serotoninergic function contribute to SDB in PD. In this study, we evaluated the relationship between caudal brainstem serotoninergic neuron integrity and SDB in PD.

## Methods

### Objectives

The primary objective of this study was to correlate caudal brainstem serotoninergic innervation *in vivo* with an objective measure of SDB. The hypothesis tested is that degeneration of caudal brainstem serotoninergic neurons contributes significantly SDB in PD. We used [^11^C]DASB PET imaging to assess caudal brainstem SERT expression in PD participants. To assess participant perceived and objective aspects of sleep function we used standardized sleep and wake rating scales, and polysomnograpy (PSG) in the same participants. As a secondary objective, we assessed the relationship between an *in vivo* measure of striatal dopaminergic denervation, the PET ligand [^11^C]dihydrotetrabenazine ([^11^C]DTBZ), a highly specific ligand for the vesicular monoamine transporter type-2 (VMAT2), and measures of SDB. In *post hoc* exploratory analyses, we examined correlations between clinical variables, PSG measures of sleep characteristics, and clinical sleep evaluations.

### Participants

PD participants were recruited from the Movement Disorders Clinic at the University of Michigan. Subjects were part of a larger cohort of subjects studied as part of a larger project studying non-motor features of PD. In general, participants in this study are non-demented PD subjects of mild to moderate severity not using confounding medications. Subjects must be willing to undergo several imaging studies and extensive clinical characterization. Our total cohort is now approximately 130 PD subjects. This cross-sectional study involved 51 participants with PD drawn from the larger group on the basis of willingness to undergo polysomnography and absence of confounding medications. Subjects were not selected on the basis of sleepiness. Inclusion criteria included PD according to the UK Parkinson's Disease Society Brain Bank Research Center clinical diagnostic criteria, modified Hoehn and Yahr stages 1 to 4, and a typical pattern of nigrostriatal dopaminergic denervation as demonstrated by [^11^C]DTBZ PET imaging [31], [32]. No participants received neuroleptics, psychostimulants, antimuscarinics, acetylcholinesterase inhibitor drugs, trazodone, modafinil, anti-depressants (selective serotonin reuptake inhibitors; SSRIs; tricyclics, TCAs), St. John’s Wort, or bupropion for at least 2 months prior to study entry. No participants were taking activating or sedating medications, including benzodiazepines and antihistamines, in the two weeks prior to PSG. No participants had signs of a stroke in a clinically relevant area (cerebral cortex, basal ganglia, thalamus), or a mass lesion on magnetic resonance brain imaging (MRI), or a history of deep brain stimulation surgery.

Each participant underwent clinical testing with recording of demographic information, neurological examination to determine the presence and severity of PD, neuropsychiatric and neurobehavioral tests, and sleep questionnaires to assess the experienced level of sleep disturbance. For objective sleep evaluation, PSG and multiple sleep latency tests (MSLTs) were obtained in all PD participants. All participants underwent [^11^C]DASB and [^11^C]DTBZ PET imaging, and structural MRI for anatomical co-registration.

### Clinical Test Battery

We recorded demographic and clinical information for each participant to obtain information about the onset of motor symptoms, cognitive symptoms, mood symptoms, medication use including L-dopa equivalents doses (LEDs), and co-morbidities. A neurological examination including modified Hoehn & Yahr staging, and the MDS-revised Unified Parkinson Disease Rating Scale (UPDRS) in the practically defined ‘off’ state was used to assess the severity of PD [33]. We used the Mini Mental State Examination (MMSE) to assess cognitive status.

### Sleep Evaluation

We used the Berlin Sleep Questionnaire (BQ), Parkinson Disease Sleep Scale (PDSS), and Epworth Sleepiness Scale (ESS) to assess participant subjective sleep quality and daytime alertness [34]–[36]. PSG was performed for objective evaluation of SDB. PSG included 6 standard EEG channels, 2 EOG channels, chin and bilateral anterior tibialis surface EMG, 2 EKG leads, nasal and oral airflow monitors (thermocouples), nasal pressure monitoring, thoracic and abdominal excursion monitor (uncalibrated inductance plethysmography), assessment of snoring, and finger oximetry. All scoring followed standard guidelines and was performed by a single, experienced registered polysomnographic technologist masked to all imaging results [37]. A board-certified sleep specialist blinded to results of PET imaging studies (RDC) reviewed scoring and interpreted each sleep study. Apneas, hypopneas, and respiratory event-related arousals (RERAs) were scored and used to calculate the respiratory disturbance index (RDI, number of events per hour of sleep) as a standard measure of sleep apnea severity [37]. Following conventional definitions, a score of 0–5 was classified as no SDB, >5–15 as mild SDB, >15–30 as moderate SDB, and >30 as severe SDB. Patients underwent PSG after taking their normal schedule of dopamine replacement medications. MSLTs were performed the morning after PSG and naps were scored for the latency between lights out and the first epoch of stage 1 sleep [38]. The mean sleep latency scores across 5 naps provided the objective measure of daytime sleepiness used in this study.

### PET and MR Imaging

All participants underwent [^11^C]DASB and [^11^C]DTBZ PET imaging for an estimate of serotoninergic and dopaminergic terminal integrity, respectively. MRI was acquired in all participants for intra-participant and intermodality image registration, and assignment of standard volumes of interest (VOIs).

### MR Imaging

Magnetic resonance imaging was performed on a 3 Tesla Philips Achieva system (Philips, Best, The Netherlands) utilizing an eight-channel head coil and the ‘ISOVOX’ exam card protocol primarily designed to yield isotropic spatial resolution. A standard T1-weighted series of a 3D inversion recovery-prepared turbo field echo was performed in the sagittal plane using repetition time/echo time/inversion time  = 9.8/4.6/1041 ms; turbo factor  = 200; single average; field of view  = 240×200×160 mm; acquired matrix  = 240×200. One hundred and sixty slices were reconstructed to 1 mm isotropic resolution. This sequence maximizes contrast among grey matter, white matter and cerebrospinal fluid and provides high-resolution delineation of cortical and subcortical structures [39].

### PET Imaging

The radiotracers [^11^C]DASB and [^11^C]DTBZ were given intravenously and participants were imaged following standard imaging procedures at the University of Michigan. [^11^C]DASB and [^11^C]DTBZ PET imaging were performed in 3D imaging mode using an ECAT HR+ tomograph (Siemens Molecular Imaging, Inc., Knoxville, TN), which acquires 63 transaxial slices (slice thickness  = 2.4 mm; intrinsic in-plane resolution  = 4.1 mm full width at half maximum over a 15.2 cm axial field of view). A NeuroShield (Scanwell Systems, Montreal, Canada) head holder/shielding unit was attached to the patient bed to reduce the contribution of detected photon events originating from the body outside the scanner field of view [40]. Prior to the [^11^C]DASB and [^11^C]DTBZ injections, a 5 minute transmission scan was acquired using rotating ^68^Ge rods for attenuation correction of emission data using the standard vendor supplied segmentation and re-projection routines. All participants were studied in the supine position, eyes and ears unoccluded, resting quietly in a dimly lit room.

### [^11^C]DTBZ Imaging

No-carrier-added [^11^C]DTBZ (250–1000 Ci/mmol at the time of injection) was prepared as reported previously [41]. Dynamic PET scanning was performed for 60 min immediately following a bolus injection of 55% of 555 mega-Becquerel (15 milliCuries) of [^11^C]DTBZ dose over the first 15–30 s of the study, while the remaining 45% of the dose was continuously infused over the next 60 min, resulting in stable arterial tracer levels and equilibrium with brain tracer levels after 30 min [42]. A series of 15 frame sequence of scans over 60 min were obtained as following: four × 30 s; three × 1 min; two × 2.5 min; two × 5 min; and four × 10 min.

### [^11^C]DASB Imaging

Dynamic PET scanning was performed for 80 min immediately following a bolus intravenous injection of 666 mega-Becquerel (18 milliCuries) of [^11^C]DASB. Radiotracer was administered as a bolus plus constant infusion using 70% as a slow bolus over 30 secs, followed by constant infusion of the remaining 30% over the 80 mins study duration. The [^11^C]DASB-PET studies were acquired as 17 scan frames over a total of 80 mins as follows: 4×30 secs; 3×1 min; 2×2.5 mins; 2×5 mins; 6×10 mins.

### Image Analysis

IDL image analysis software (Research systems, Inc., Boulder, CO) was used to trace volumes of interest (VOIs) manually on the T1-weighted MRI scan, representing the caudate, putamen, medulla oblongata, pons, and inferior posterior cerebellar cortex. Cortical volumes of interest were defined using semi-automated threshold delineation of the cortical grey matter signal. All PET image frames were spatially co-registered within participants with a rigid-body transformation to reduce the effects of participant motion during the imaging session [43]. These motion-corrected PET frames were spatially co-registered to the T1-weighted MRI using standard co-registration procedures in SPM8b implemented in Matlab R2010b (MathWorks). Time activity curves for each VOI were generated from the spatially aligned PET frames. [^11^C]DASB SERT and [^11^C]DTBZ VMAT2 distribution volume ratio (DVR) was estimated using the Logan plot graphical analysis method with the regional time activity curves as the input function. Specific binding potential (BP) was calculated by subtracting 1 from the DVR to estimate the density of available receptors [44]. The inferior posterior cerebellar cortex was used as the reference region for [^11^C]DASB and the neocortex as reference region for [^11^C]DTBZ [42], [45]–[48]. For analysis of [^11^C]DASB data, single regional BPs for each participant were derived by averaging regional BPs from each side of the brainstem. Our prior research indicates bilateral and symmetric degeneration of serotoninergic projections [29]. In PD, asymmetrical striatal dopaminergic terminal loss is observed, therefore the striatal [^11^C]DTBZ BPs were divided into most affected (MA) and least affected (LA) sides for each participant [39].

### Ethics

The study was approved by the University of Michigan Medical School Institutional Review Board for Human Participant Research (IRBMED). Prior to participation, all participants received, discussed, and signed a written informed consent approved by the IRBMED.

### Statistical Analysis

To test our hypothesis, we performed linear regression analysis using the bilaterally averaged [^11^C]DASB caudal brainstem (pons and medulla) BPs, [^11^C]DTBZ striatal BPs, LEDs, and BMIs as regressors with RDI as the dependent variable. For an effect size of 0.35, α = 0.05, power  = 0.9, and 4 predictors, N = 45. In *post hoc* exploratory correlative analyses, we assessed which clinical variables correlated with PSG measures of SDB using Spearman rank order correlations. We evaluated possible relationships between RDI and min O_2_ and age, BMI, UPDRS scores, LEDs, and results of sleep questionnaires. In these analyses, we controlled for BMI, age, and LED by using partial correlations. We controlled for multiple comparisons with False Discovery Rate (FDR) adjusted p values at a maximally acceptable level of 0.05.

## Results

### Clinical Findings

Of 51 participants, 43 were male and 8 were female. Forty nine participants were on dopamine replacement therapy: 26 participants received L-dopa therapy, 4 participants received a dopamine agonist, and 16 participants received a combination of L-dopa and a dopamine agonist. Ten of these participants received a MAO-inhibitor and 6 amantadine. Two participants received only a MAO-inhibitor and 1 participant received only amantadine. Two participants received no therapy. Mean levodopa equivalent dose (LED) was 772 (SD 492) [49]. Hoehn and Yahr staging showed that most participants exhibited mild to moderate disease severity: 11 participants stage 1.5, 12 participants stage 2, 26 participants stage 2.5, and 2 participants stage 3. Participants had mean age of 63.9 years (SD 6.2) and had mean duration of the disease of 6.0 years (SD 3.7). Almost all participants had MMSE scores in the normal range with mean 29 (SD 1). Mean participant BMI was 28 (SD 4). Mean PDSS scores were 110 (SD 18) and mean ESS scores were 9 (SD 5). Participant characteristics are summarized in Table 1.

**Table 1:** Participant Characteristics.

	Distribution		Mean (SD)		
**Gender**	43M/8F		–		
**Age (years)**	50–84		64 (6)		
**Disease Duration (years)**	1–14		6 (4)		
**Hoehn & Yahr stage**	1.5–3		2.2 (0.4)		
**UPDRS total**	15–54		32 (10)		
**LED**	0–2098		772 (493)		
**MMSE**	22–30		29 (1)		
**BMI**	19–42		28 (4)		
**PDSS**		65–142		110 (18)	
**ESS**		1–21		9 (5)	
**BQ (low risk/high risk)**		28/23		–	

SD  =  Standard Deviation; M  =  Male; F  =  Female; UPDRS  =  Unified.

Parkinson Disease Rating Scale; LED  =  Levodopa Equivalent Dose;

MMSE  =  Mini Mental State Examination; BMI  =  Body Mass Index;

PDSS  =  Parkinson’s Disease Sleepiness Scale; ESS  =  Epworth.

Sleepiness Scale, BQ  =  Berlin Questionnaire.

### PSG Characteristics

PSG data revealed that on average, PD participants had moderate SDB, with a mean RDI of 23 (SD 20). Participants were distributed broadly among normal, mild, moderate, and severe SDB categories. Twelve subjects had normal PSGs (24%) and the remainder exhibited SDB (76%); 6 had mild SDB, 20 had moderate SDB, and 13 had severe SDB. Most of the apneic events consisted of hypopneas. Mean min O_2_ was 87% (SD 5) and min O_2_ correlated well with total RDI (ρ = −0.448; p<0.01), RDI-obstructive apneas (ρ = −0.567; p<0.01), and RDI-hypopneas (ρ = −0.49; p<0.01). PSG results are summarized in Table 2.

**Table 2:** Mean (SD) of sleep parameters and the number of events on the Respiratory Disturbance Index.

	Mean (SD)
**Total recording time (min)**	486 (17)
**Total sleep time (min)**	352 (77)
**Stage N1 (% of total sleep time)**	23 (19)
**Stage N2 (% of total sleep time)**	56 (16)
**Stage N3 (% of total sleep time)**	6.1 (6.7)
**State R (% of total sleep time)**	15 (7.9)
**Arousals/hr**	27 (17)
**Sleep efficiency (% of total sleep time)**	73 (15)
**RDI-Total**	23 (20)
**RDI-Central Apneas**	1.2 (2.4)
**RDI-Obstructive Apneas**	3.1 (7.6)
**RDI-Hypopneas**	16 (14)
**RERAs**	3.0 (3.6)
**Min O_2_**	88 (3.4)

SD  =  Standard Deviation; RDI  =  Respiratory Disturbance Index; RERA  =  Respiratory Effort Related Arousal; Stage N1–N3 =  non-REM stage 1–3; Stage R  =  REM stage; Min O**_2_**  =  Minimal oxygen saturation; Arousals/hr  =  Arousals per hour of sleep.

### [^11^C]DASB & [^11^C]DTBZ PET Imaging Results

The distribution of [^11^C]DASB binding resembled those of prior [^11^C]DASB PET studies [27]–[30]. The highest mean BPs were found in the raphe nucleus with intermediate binding in several structures of the basal ganglia (caudate, striatum, putamen, and substantia nigra), thalamus and amygdala, and caudal BPs in the hippocampus, medulla, pons, and lowest in the neocortex (data not shown). Mean medullary BP was 0.59 (SD 0.17) and mean pontine BP was 0.70 (SD 0.16). Striatal [^11^C]DTBZ binding exhibited a mean BP of 0.95 (SD 0.29). The LA caudate and putaminal BPs were 1.12 (SD 0.33) and 0.94 (SD 0.43), respectively, with the MA caudate and putaminal BPs 1.04 (SD 0.28) and 0.69 (SD 0.23), respectively. As described previously, there was an anterior to posterior gradient with the caudal putamen most affected and the caudate relatively spared (data not shown) [39].

### Relationship between PSG Measures of SDB and PET Imaging Findings

**Figure 1:**
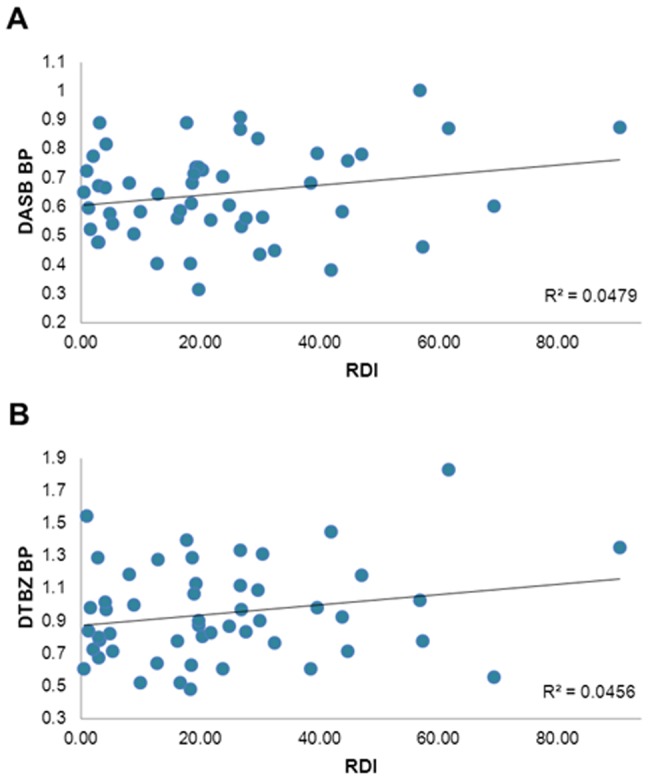
Correlations between participant RDIs and A) [^11^C]DASB caudal brainstem BPs; B) [^11^C]DTBZ striatal BPs.

Regression analysis to predict RDI with bilateral caudal brainstem [^11^C]DASB BPs, bilateral striatal [^11^C]DTBZ BPs, LEDs, and BMIs as predictor variables showed an overall significant effect (F = 2.85, p  = 0.03); RDI was predicted by BMI (t = 2.10, p = 0.04), however, not by caudal brainstem [^11^C]DASB BP (t = 1.06, p  = 0.29), striatal [^11^C]DTBZ BP (t  = 0.83, p  = 0.41), or LED (t = −1.49, p  = 0.14). Figure 1 shows scatterplots of the relationships between RDI and caudal brainstem [^11^C]DASB binding (Figure 1A), and striatal [^11^C]DTBZ binding (Figure 1B). To reduce a potential floor effect due to severe dopaminergic denervation, bilateral striatal [^11^C]DTBZ binding was replaced with unilateral striatal [^11^C]DTBZ binding corresponding to the clinically least affected body side. This did not significantly change the model (analysis not shown).

### Exploratory Analysis: Polysomnography, Clinical, and Sleep Scale Correlations

BMIs correlated significantly with rates of obstructive apneas (rho  = 0.385, p  = 0.005) and rates of hypopneas (rho  = 0.386, p  = 0.005). MSLTs correlated significantly with total RDI (rho = −0.361, p = 0.009) and with latency to nocturnal sleep (rho = 0.546, p = 0.000). Some clinical features correlated well with the objective sleep data (Table 3). One strong finding was that higher UPDRS scores, an indication of PD severity, correlated inversely with sleep efficiency (rho = −0.460, p = 0.001) and more time awake after sleep onset (rho = 0.314, *p* = 0.025). Even after controlling for BMI and age, significant correlations between total UPDRS scores and sleep efficiency and time awake after sleep onset persisted. Also after controlling for LED, correlations between the total UPDRS scores and sleep efficiency (rho = −0.476, p = 0.00) as well as time awake after sleep onset (rho = 0.44, p = 0.001) were significant. UPDRS scores did not correlate with any of the RDI measures (Table 3). Sleep measures such as the ESS and PDSS showed no significant associations with the PSG measures after multiple comparisons correction (Table 4).

**Table 3:** Spearman correlation coefficients between clinical variables and sleep variables.

	Age	BMI	UPDRS-Total	LED
**Sleep Efficiency**	−0.212	0.007	−0.460*	0.002
**Wake after sleep Onset**	0.213	−0.011	0.399*	−0.037
**Sleep Latency**	−0.013	−0.114	0.314	−0.074
**RDI**	−0.074	0.295	0.039	−0.156
**RDI-Obstructive Apneas**	−0.003	0.385*	0.090	−0.256
**RDI-Central Apneas**	−0.116	−0.095	−0.217	−0.160
**RDI-Hypopneas**	−0.032	0.388*	0.026	−0.203
**RDI-RERA**	−0.227	0.216	−0.098	−0.035

* Significant after FDR correction; BMI  =  Body Mass Index; UPDRS  =  Unified Parkinson.

Disease Rating Scale; RDI  =  Respiratory Disturbance Index; RERA  =  Respiratory Effort Related Arousal.

## Discussion

We aimed to determine whether SDB in PD is related to caudal brainstem serotoninergic neuron degeneration by measuring caudal brainstem SERT binding with [^11^C]DASB PET imaging and quantifying SDB with PSG. We further assessed the relationship between striatal dopaminergic degeneration in PD participants using [^11^C]DTBZ PET imaging and PSG results. Although we found both a high frequency of SDB in PD and significant variation in brainstem [^11^C]DASB binding, caudal brainstem [^11^C]DASB binding did not predict SDB severity (as measured by RDI) in our PD participants. These results are inconsistent with a clear role for serotoninergic neuron degeneration in the genesis of SDB in PD. Similarly, there was no correlation between changes in striatal [^11^C]DTBZ binding and PSG measures of SDB. Since we found no significant associations between [^11^C]DASB or [^11^C]DTBZ binding and PSG measures, changes in other neuromodulatory systems may play a more significant role in the genesis of SDB and sleep disruption in PD participants.

Other neuromodulatory systems that could influence SDB observed in PD are the noradrenergic (NE) or the cholinergic (ACh) neuronal systems. Significant degeneration of the locus coeruleus, the source of NE innervation, has been observed in PD patients [50]. Similar to serotonin neurons of the caudal brainstem, NE neurons regulate brainstem motoneurons involved in maintaining upper airway patency and respiration [51], [52]. Reticular cholinergic neurons may also regulate relevant brainstem motoneurons [53], [54]. Several studies have shown cholinergic projection systems degeneration in PD patients [55], [56]. Among patients with Multiple System Atrophy, which often exhibits Parkinsonian features, degeneration of brainstem cholinergic projections is suggested as a possible cause of SDB [57].

**Table 4:** Spearman correlation coefficients between sleep variables from polysomnography and subjective sleep measures.

	Epworth	PDSS
**Sleep Latency**	−0.351	0.014
**REM Latency**	0.094	−0.112
**Wake after sleep onset**	−0.029	−0.107
**Min O_2_**	−0.193	0.060
**Mean MSLT**	−0.370	0.033
**Sleep Efficiency**	0.112	0.110
**RDI**	0.199	0.110

None significant after multiple comparisons correction.

Epworth  =  Epworth sleepiness scale; PDSS  =  Parkinson’s Disease Sleep Scale;

REM  =  Rapid Eye Movement sleep; Min O_2_  =  Minimum Oxygen Saturation;

MSLT  =  Multiple Sleep Latency Tests; RDI  =  Respiratory Disturbance Index.

There are some potential limitations of our results. Our approach assumes that SERT binding measured with [^11^C]DASB PET imaging reflects integrity of serotoninergic terminals. It is possible that some component of differences in SERT binding between participants could be due to differential regulation of SERT expression. Because loss of serotoninergic neuronal perikarya is well documented in post-mortem studies of PD participants, attributing differences in SERT binding to differential loss of serotoninergic neurons and terminals is the most parsimonious explanation for differences in regional [^11^C]DASB binding in our participants [26], [58], [59]. To avoid participant discomfort, PSGs were performed with participants on their usual dopamine regimen. This could obscure a relationship between PSG results and striatal [^11^C]DTBZ binding results.

There is some disagreement about the prevalence and clinical significance of SDB in PD. While a number of clinic series report increased prevalence of SDB in PD, some recent series suggest that SDB prevalence is not higher than that of control populations [12], [14], [15], [60], [61], [62]. No study of SDB in PD to date, however, has been conducted with true population based samples. Our finding of a high frequency of SDB (as judged by PSG criteria) in a series of PD participants not selected for sleepiness is most consistent with increased prevalence of SDB in PD. It has also been suggested that SDB in PD, particularly when detected by PSG, is of little clinical import [12]. This may be correct, particularly for individuals with mild SDB on PSG. Even mild SDB, however, could be significant in the context of PD. Depressive symptoms and/or cognitive impairment contribute significantly to impaired quality of life in PD and co-morbid SDB could significantly exacerbate these important features of PD. Regardless of the clinical significance of SDB in PD, the apparent high prevalence of SDB in PD suggests an interesting biological difference in regulation of upper airway function and control of respiration during sleep in PD.

There were correlations between clinical features of PD and some PSG data. Higher total UPDRS scores correlated well with decreased sleep efficiency, time awake after sleep onset, and increased sleep latency. These results suggest a relationship between PD disease severity, and sleep quantity and quality. Higher BMI as well as minimum oxygen saturation correlated well with RDI, especially with obstructive apnea and hypopneas. This finding is not surprising as increased BMI is a major risk factor for SDB.

Clinical sleep measures (PDSS and ESS) did not show strong correlations with most of the objective sleep variables measured by PSG/MSLT. These findings are consistent with recent studies by Noradina *et al.* (2010) and Norlinah *et al.* (2010) in which there was no relationship between PDSS and PSG measures in 46 PD participants studied [15], [60]. Trotti and Bliwise (2010) found no correlation between ESS and PSG measures of SDB in PD participants [61]. In our data, increased sleepiness as measured with the ESS did not correlate with sleep latencies on both PSG and MSLTs. PSG studies, however, provide only a snapshot of sleep dysfunction while rating scales assess sleep dysfunction on longer time-scales (2–3 weeks). In non-PD participant samples, the ESS shows little correlation with PSG measures of SDB or MSLTs [63]. Prior studies and our data suggest a complicated relationship between clinical sleep scale measures and PSG/MSLT results in PD participants.

We can conclude that neither serotoninergic nor dopaminergic neuron degeneration in PD are likely to play a major role in the development of SDB observed in PD patients. We suggest that another neuromodulatory system might play a more pronounced role in developing SDB in PD and that investigating this issue might improve understanding of underlying neuromodulatory systems involved in non-motor symptoms of PD.
